# Electromechanical Photophysics of GFP Packed Inside Viral Protein Cages Probed by Force-Fluorescence Hybrid Single-Molecule Microscopy

**DOI:** 10.1002/smll.202200059

**Published:** 2022-06-19

**Authors:** Klara Strobl, Ekaterina Selivanovitch, Pablo Ibáñez-Freire, Francisco Moreno-Madrid, Iwan A. T. Schaap, Rafael Delgado-Buscalioni, Trevor Douglas, Pedro J. de Pablo

**Affiliations:** Department of Condensed Matter Physics, Universidad Autónoma de Madrid, Madrid 28049, Spain; Department of Chemistry, Indiana University, Bloomington, IN 47405, USA; Department of Condensed Matter Physics, Universidad Autónoma de Madrid, Madrid 28049, Spain; Department of Condensed Matter Physics, Universidad Autónoma de Madrid, Madrid 28049, Spain; Hensoldt Optronics GmbH 35576 Wetzlar, Germany; Department of Condensed Matter Physics, Universidad Autónoma de Madrid, Madrid 28049, Spain; Institute of Condensed Matter Physics (IFIMAC), Universidad Autónoma de Madrid, Madrid 28049, Spain; Department of Chemistry, Indiana University, Bloomington, IN 47405, USA; Department of Condensed Matter Physics, Universidad Autónoma de Madrid, Madrid 28049, Spain; Institute of Condensed Matter Physics (IFIMAC), Universidad Autónoma de Madrid, Madrid 28049, Spain

**Keywords:** atomic force microscopy, compartmentalization, correlative microscopy, molecular crowding, single-molecule manipulation, total internal reflection fluorescence microscopy, virus cages

## Abstract

Packing biomolecules inside virus capsids has opened new avenues for the study of molecular function in confined environments. These systems not only mimic the highly crowded conditions in nature, but also allow their manipulation at the nanoscale for technological applications. Here, green fluorescent proteins are packed in virus-like particles derived from P22 bacteriophage procapsids. The authors explore individual virus cages to monitor their emission signal with total internal reflection fluorescence microscopy while simultaneously changing the microenvironment with the stylus of atomic force microscopy. The mechanical and electronic quenching can be decoupled by ≈10% each using insulator and conductive tips, respectively. While with conductive tips the fluorescence quenches and recovers regardless of the structural integrity of the capsid, with the insulator tips quenching only occurs if the green fluorescent proteins remain organized inside the capsid. The electronic quenching is associated with the coupling of the protein fluorescence emission with the tip surface plasmon resonance. In turn, the mechanical quenching is a consequence of the unfolding of the aggregated proteins during the mechanical disruption of the capsid.

## Introduction

Molecular function at the subcellular level is highly determined by confinement and mechanical cues. Many proteins work in living systems while being pushed and squeezed by other biomacromolecules.^[1]^ On the one hand, molecular crowding modifies the dynamics of the proteins’ conformational changes due to excluded volume effects.^[1]^ On the other hand, it favors protein aggregation, which can affect their function^[2,3]^ and might have important biological consequences.^[4,5]^ In vivo sequestration of proteins in cellular compartments^[1,6]^ such as bacterial microcompartments, carboxysomes, or the endoplasmic reticulum^[7]^ induces molecular crowding with concentrations of ≈100 mg mL^−1^. This is in contrast with standard experiments in dilute solutions (1 mg mL ^−1^)^[8]^ and consequently, it is likely that some differences may arise between the function of the molecules in these distinct environments.^[9]^ Molecular crowded environments within the cell are mimicked in molecular ensembles using artificial crowding agents,^[10]^ for example, PEG, Ficoll, and triton, that can induce undesired effects, such as aggregation or binding.^[11,12]^ In this context, the development of nanoscale molecular containers^[13–15]^ imitates the fundamental biological problems regarding compartmentalization^[16,17]^ with promising technological applications.^[18]^ Encapsulation protects molecules from harsh environments^[19]^ and in contrast to systems of free molecules,^[10]^ the packing fraction determines the cargo microenvironment, thus minimizing the influence of external factors. Molecular containers offer the possibility of isolating and studying the effects of confinement on specific molecules at the mesoscale, providing insights into their intracellular behavior as well as the design of enzymatic systems for controlled catalysis.^[20]^ One key issue is to determine how the confinement of molecules affects their function and performance under challenging conditions. Several effects can occur including alterations in their diffusion rate, conformational changes, and stability under variations in their microenvironment.^[21]^

The structural simplicity and robustness of self-assembled viral capsids has led to the use of well-characterized and highly homogeneous virus-like particles (VLPs) to synthesize nano-compartments with control over size, packing density, and location of the encapsulated molecules.^[22]^ VLPs derived from the *Salmonella typhimurium* bacteriophage P22 are suitable models which can be used to address some of these questions, as this is a versatile and widely studied system in virology and nanomaterial synthesis.^[23]^ The P22 VLP capsid comprises 420 copies of a coat protein (CP) that assembles into a *T* = 7 icosahedral shell with the aid of up to 300 copies of an internalized scaffolding protein (SP).^[24]^ Truncated SP interacts with CP by an helix-loop-helix motif^[25]^ so that non-native cargo molecules can be genetically fused to either of the N- or C-termini.^[15,26]^ By contrast to infectious phages, these engineered VLPs have 12 identical pentons with no portal^[27]^ resulting in a higher symmetrical structure that enables a homogeneous distribution of the cargo. Leveraging the P22 VLP self-assembly mechanism and the role of SP, a variety of proteinaceous cargo molecules have successfully been encapsulated using both in vivo and in vitro approaches, with the latter affording compositional control of the encapsulated cargo.^[28,29]^ In the particular case of the green fluorescent protein (GFP) cargo, in vitro purification enables the regulation of the quantity of packed proteins.

Molecular crowding studies of confined and non-confined molecular systems are normally carried out on a large number of molecules (≈N_A_).^[10,30]^ These ensemble approaches provide the average behavior of the molecules in the test tube.^[6,26]^ Although this mean value could be assumed to be the canonical result of a single structure, the asynchronous behavior of each molecule^[12,31]^ may hinder intermediate states and impair the correct interpretation of the experiments.^[32]^ In addition, the study of different situations, such as the crowding agent concentration, requires distinct experiments to reach static and homogeneous conditions throughout the molecular ensemble.^[6]^ Therefore, these ensemble measurements make it difficult to tune the microscopic environment of proteins back and forth in real time, and various samples need to be prepared to explore diverse conditions.^[11]^ Even in the case of nanocontainers, the study of different packing situations requires the preparation of new nanocages with a distinct loading fraction. This fact hinders changing the microenvironment of the same particle in situ. By contrast, single molecule techniques are extensively used to probe events in kinetics and structure that overcome ensemble-averaged properties.^[33]^ Furthermore, single-molecule manipulation using force strokes such as atomic force microscopy (AFM)^[31]^ can be applied to tailor the microenvironment of individual molecular containers^[24]^ and change the cargo’s physical conditions in real time. AFM-fluorescence correlative microscopy enables the study of fluorescent-labeled biological samples^[34,35]^ by imaging the emission signal and the topography of the same region. Beyond mere passive imaging, further developments of this approach aim to monitor the molecular function dynamics of the sample under active (physical) manipulation in real time, such as bacterial death^[35]^ and DNA diffusion from cracked viruses.^[36]^

In this research we have packed GFPs within P22 procapsids as a model system for investigating the effects of confinement and physical perturbation on its ability to radiate light. GFP emission peaks roughly at ≈510 nm (green) after being excited at ≈489 nm (blue)^[37]^ and it is extensively used as a biological marker.^[38]^ GFP is subjected to crowded environments in nature when confined at plasma membranes.^[39]^ Beyond its evident biotechnological interest, it has recently been reported that the emissive state requires the complete integrity of its structure.^[40]^ In this work, we have studied the photophysical behavior of individual GFP-loaded P22 nanocages under a microenvironment change induced by an AFM tip. The fluorescence signal of single VLPs, measured while probing the nanoparticle structure in real time has revealed reversible quenching effects of an electromechanical origin.

## 2. Results

### 2.1. Packing GFP within the P22 Prohead

**Figure 1.**
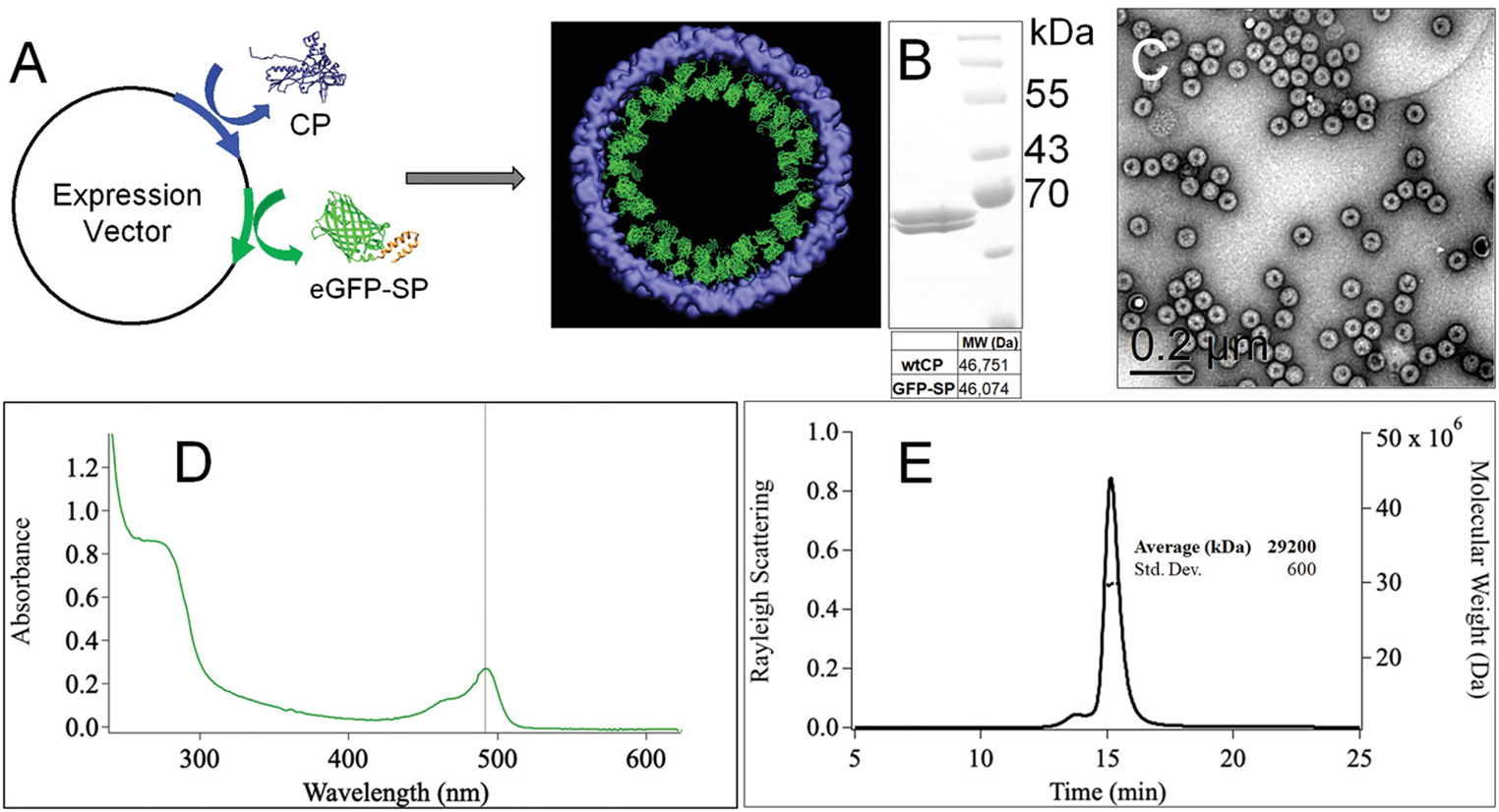
Synthesis and biochemistry of GFP-loaded P22 VLPs. A) Left, The co-expression of coat protein (CP, blue) with N-terminal truncated scaffold protein (SP, orange) fused to GFP (green) monomers lead to VLP assembly of procapsid with encapsulated eGFP. Right, a cross section of the coarse-grained modeled (Experimental Section) virus capsid (purple) with 224 GFPs (green). B) A SDS-PAGE denaturing gel showing the wtCP and GFP-SP proteins at the double band on the left. C) TEM image of P22-GFP particles with expected morphology and 56 nm diameter. D) The UV–vis spectrum of P22-GFP particles with a characteristic GFP absorbance peak at 492 nm. E) SEC-MALS of P22-GFP Rayleigh scattering with the average MW of the particles.

Truncated SP directs the assembly of P22 procapsid VLPs (56 nm diameter, 46 × 10^3^ nm^3^ internal volume) and can be genetically fused to other proteins resulting in their encapsulation into the particles. This approach was used to incorporate the GFP (PDB: 2Y0G) into the P22 interior via the expression of CP and GFP-SP fusion (Figure 1A and Experimental Section).^[28,29]^ The individual gene products were identified in purified P22-GFP particles using denaturing gel electrophoresis (Figure 1B), which produced two bands corresponding to the expected molecular weights of both CP and GFP-SP. Transmission electron microscopy images (Figure 1C) showed the expected size (56 nm) and morphology of the particles. In a previous study using cryo-EM reconstructions, we determined the location of the GFP at the virus lumen as a subshell just below the virus capsid by comparing full and empty P22 procapsids.^[24]^ The number of fluorescent cargo molecules was calculated from the MW measured using multi-angle light scattering coupled with size-exclusion chromatography (SEC-MALS) and verified using UV–vis (Figure 1D,E).

By using an in vivo approach^[28]^ the particles packed the maximum number of GFPs, achieving an average of ≈209 GFP-SP molecules organized around the internal lumen,^[41]^ with a packing fraction of 36% and a relatively unoccupied center (Figure 1A, right). The internalization of GFPs has also been demonstrated by measuring virus mechanics.^[24]^ In this study, we also observed that the VLP size found in cryo-EM and AFM data correspond to the diameter of wild type P22 procapsids. The fluorescence lifetimes of the free GFP-SPs and those encapsulated within the P22 procapsids were comparable at 3.05 and 2.85 ns, respectively, suggesting that the packaging of these molecules had little influence on their intrinsic fluorescence (Supporting Figure S1).

### 2.2. Combining Single-Molecule Fluorescence with High-Resolution AFM

**Figure 2.**
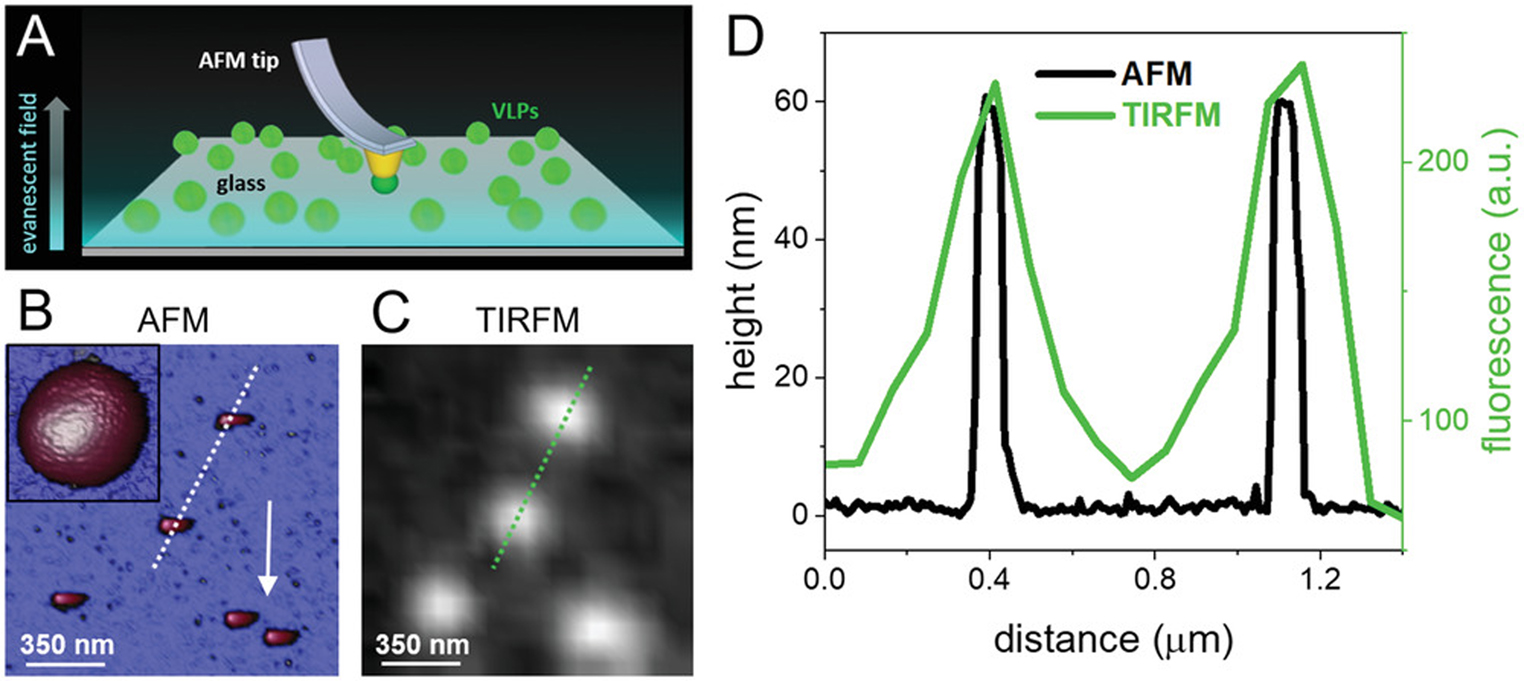
AFM-TIRFM of P22 VLPs. A) Presents a stylized diagram of the experimental system, showing the P22 VLPs (light green) and the AFM probed particles in dark green. B,C) Simultaneous AFM and fluorescence images, respectively, of individual VLPs on the glass surface. The arrow in (B) indicates a couple of particles appearing as one in fluorescence (C). The inset in (B) shows a high-resolution AFM image of P22 VLP resolving the proteinaceous structure of the virus. In the AFM image (B), the particles appear elongated on the right side due to the parachuting of the tip at the downhill part of the virus when scanning from left to right. D) Comparison of the lateral resolution of both techniques by plotting the signal profile of the AFM topography (black) and the fluorescence (green) obtained at (B) indicated by the dashed white line, and (C) indicated by the dashed green line.

Figure 2A presents the philosophy of our experiments. Individual P22 VLPs were probed by approaching the AFM tip. To simultaneously register the light during AFM manipulation experiments, we coupled a single-molecule fluorescence microscope to our AFM optimizing the optomechanical design by reducing the mechanical loop to minimize the noise of the AFM measurements.^[42]^ Both the camera and the excitation laser were mechanically and thermally isolated from the AFM head to limit heat and vibration transmission.^[36]^

Figure 2B shows that in this configuration P22 VLPs produced in vivo can be routinely imaged in a liquid milieu without altering their topography, even resolving the virus protein structure (inset, Figure 2B).^[24]^ To ensure that only the GFP-loaded P22 VLPs adsorbed on the glass surface were excited, we used a total internal reflection fluorescence (TIRF) design. This layout was necessary to minimize the background signal from the particles in suspension and the AFM probe itself, which remained largely out of the evanescent excitation field.^[43]^ This combined setup enabled a correlative identification of the VLP patterns in AFM and in TIRFM (Figure 2B,C). The diffraction-limited resolution of our optical system restricted the size of the smallest spot to *λ*/2 (≈250 nm), which is larger than the P22 VLPs. Consequently, the particles that were too close together (arrow, Figure 2B) appeared as a single fluorescence spot (bottom right, Figure 2C). Therefore, the AFM images have provided the most accurate topographic information of the individual P22 proheads. In particular, Figure 2D shows the topographic (black) and light (green) profiles of two VLPs (Figure 2B,C, dotted lines) where the particle width in the fluorescence signal is ≈500 nm, approximately five times larger than in the AFM topography. Although the lateral dimension of the AFM data is still larger than the diameter of P22 due to tip-dilation effects,^[44]^ the height of the VLP’s topographic profile in the vertical axis *z*, is in agreement with the actual P22 dimensions. Therefore, the height of the VLPs can be considered as an indicator of the structural integrity of the capsid. Sometimes single molecule fluorescence is not directly additive,^[45]^ as it strongly depends on the fluorophore used.^[26]^ Structures smaller than intact viruses also fluoresce with similar intensity. In particular, the unpacked GFPs emit as much signal as the intact particles as long as they remain attached to the surface (Figure S2A). In the AFM image (Figure S2B), particles 9 and 11 appear to be debris from broken VLPs with a height of 10 nm, but their signal is comparable to particle 6, which shows a height corresponding to the size of an intact VLP (≈60 nm, Figure S2C). This may be explained by the presence of residual structures corresponding to particle debris that were already in the solution before adsorption. Nevertheless, a statistical analysis of the fluorescence signals revealed a homogeneous population in the fluorescence emission (Figure S2D), from most of the particles.

### 2.3. Altering the Microenvironment of GFP Nanocontainers with the AFM Probe

#### 2.3.1. VLP Indentation

**Figure 3.**
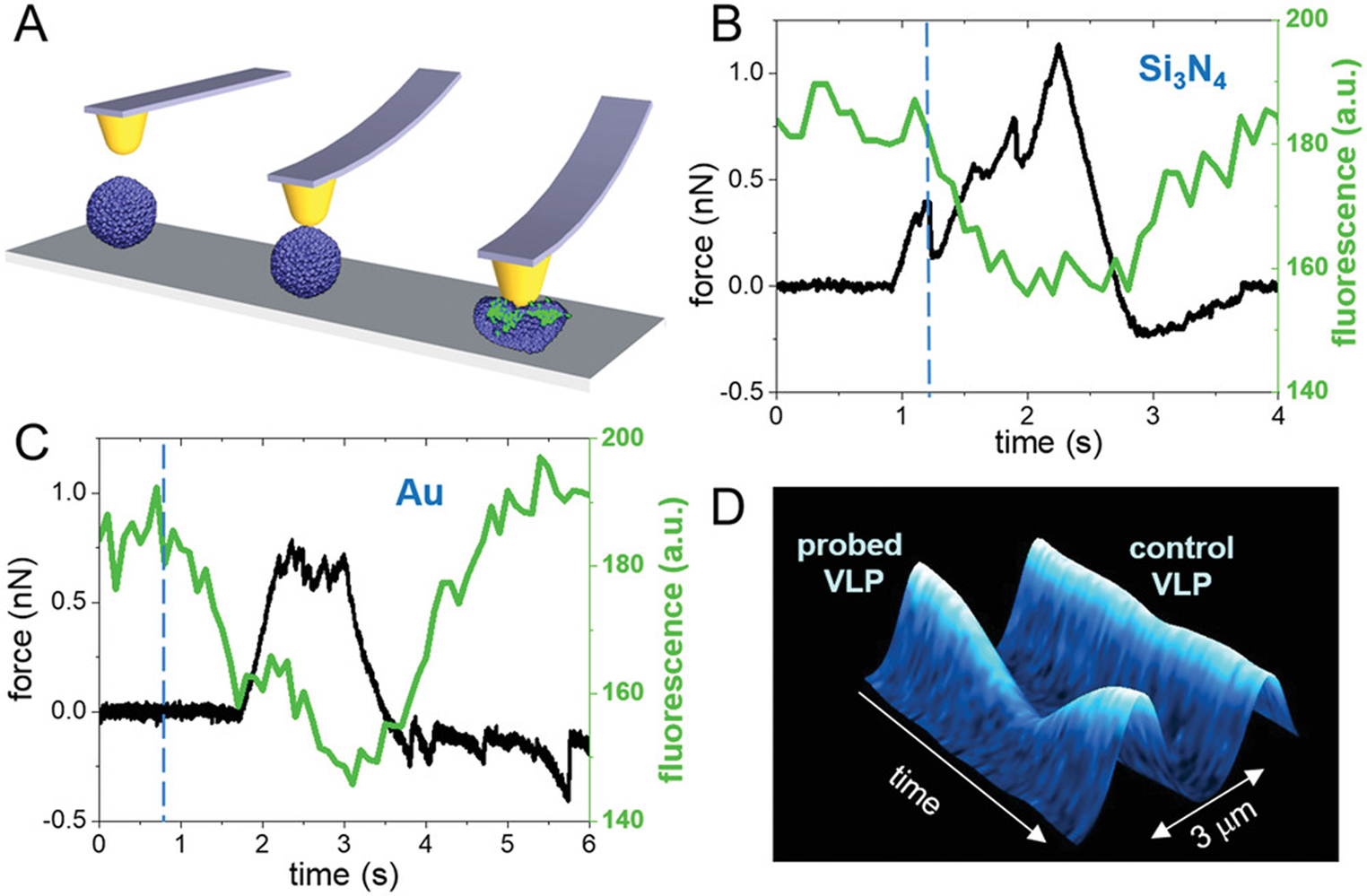
Probing the fluorescence signal of individual VLPs. A) Diagram of the AFM indentation experiment showing the three stages of the process, from non-contact (left) to virus collapse (right). B,C) The combined data charts of the synchronously measured force (black) and light (green) for Si_3_N_4_, and Au-covered tips, respectively. The blue vertical dashed lines indicate the moment at which the fluorescence decrease began (*I*_0_, Figure S5). D) Kymograph of the fluorescence evolution of the probed and control particles obtained simultaneously during the experiment, demonstrating that only the signal from the probed particle was changing.

Once a convenient P22 VLP had been located on the surface by AFM imaging, we performed a force versus distance measurement (FZ) at the very top of the structure.^[46]^ In this experiment, the AFM tip approached the virus cage vertically from a distance of ≈100 nm. At this stage (Figure 3A, left) no force was applied to the VLP and the AFM cantilever did not bend (Figure 3B, 0–1 s, and Figure 3C, 0–1.6 s, black). However, after mechanical contact had been established, the cantilever flexed and began to deform the particle (Figure 3A, middle). As the force increased, the virus container yielded, and the FZ showed a non-linear stepwise regime (Figure 3B,C, black) that corresponded to the unzipping events between the capsomers.^[46–48]^ This caused the fracture between some VLP subunits^[49]^ that were able to recover their spherical shape following the removal of the tip.^[50–53]^

#### 2.3.2. Simultaneous AFM and Fluorescence

To simultaneously register the variation of the particle’s fluorescence signal, the excitation laser was switched on only during the FZ experiment to reduce any photobleaching effects as much as possible. In this way we obtained the fluorescence evolution not only from the stressed VLP, but also from neighboring particles that could be used as a control to check for possible artifacts. Combining these microscopic techniques was a challenging problem in order to avoid crosstalk while performing simultaneous measurements.^[54]^ Figure 3D represents the kymograph of both the probed (left) and control (right) VLPs, separated by 3 μm (Supporting video SV1). These measurements show that the fluorescence quenching was caused solely by the AFM tip. In order to monitor the fluorescence evolution while the VLP was probed (Figure 3B, green), the light signal that had been registered during the FZ was superimposed on the force data (Figure 3B, black). Photob leaching has been removed as indicated in Figure S3.

#### 2.3.3. Insulator Si_3_N_4_ Tip

When probing the VLP cages with Si_3_N_4_ tips the fluorescence remained constant (Figure 3B) during tip approach (Figure 3A, left). Furthermore, the light signal stayed stable even when the tip established contact with the VLP at 1 s (Figure 3B) and linearly deformed the VLP structure (Figure 3A, center). Fluorescence quenching started at 1.2 s, coinciding with the force steps that indicated the yielding and/or breaking of the protein shell, reaching a minimum value once the particle stopped collapsing (2 s). At 2.6 s the AFM tip moved backwards, away from the surface, and the fluorescence signal remained stable until the tip was released from the VLP (3 s), and returned to a similar value to the initial magnitude (4 s).

#### 2.3.4. Conductive Au-Covered Tip

In order to manipulate the microenvironment of the VLPs in complementary ways to understand the origin of quenching, we also used Au-covered AFM tips (Experimental Section). In this case (Figure 3C), the fluorescence signal started quenching before the tip touched the VLP (≈1 s) and reached a plateau as soon as the tip made contact with the VLP (2 s). This plateau remained steady during the elastic deformation of the VLP (Figure 3A, middle), but quenching restarted coinciding with the force steps (Figure 3C, 2.3 s) indicating the mechanical rupture of the VLP (Figure 3A, right). Following this, as also seen when using the Si_3_N_4_ tip, the fluorescence recovered to its original magnitude after the tip was released from the VLP. The difference in behavior of the Au (conductive) and Si_3_N_4_ (insulator) tips highlighted the possibility that multiple mechanisms for GFP quenching might be occurring.

### 2.4. Probing VLPs Recurrently: The Structural Dependence of Quenching

We also explored the interplay between the structural state of the VLPs and their fluorescence emission. We repeated consecutive FZs on the same particle in order to induce progressive and permanent structural damage until the VLP architecture reached a collapsed conformation of ≈10 nm in height.^[55]^ Performing consecutive FZs entailed long exposure times (≈50 s) to the excitation laser. In order to calculate the effect of the structural damage, photobleaching was subtracted and the fluorescence in each FZ was normalized to the value of *t* = 0 s (Experimental Section and Figure S3).

#### 2.4.1. Conductive Au-Covered Tip

**Figure 4.**
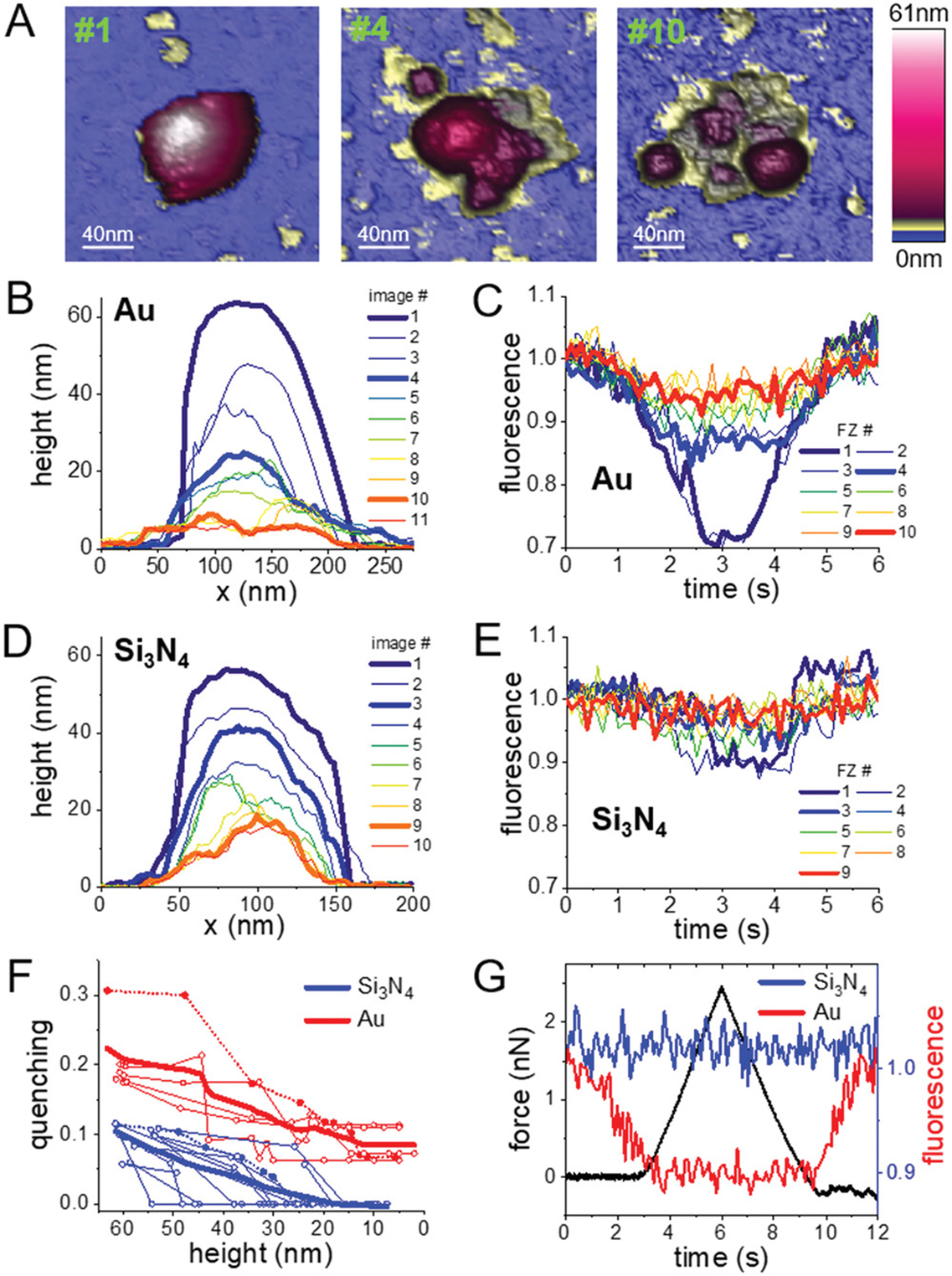
The influence of the protein cage structure on the fluorescence signal. A) AFM topographies of the same VLP through the disruption process induced by ten consecutive FZs (indentations) performed with a gold covered tip: FZ#1 is performed on image #1, FZ#4 on image #4, and FZ#10 on image #10. B) Sequential topographical profiles after performing the corresponding FZs. The thick plots correspond to the topographies of (A). The particles appear distorted in the AFM images displayed in (A) due to tip-dilation effects, thus we have focused on the height which is unaffected by the probe’s shape. C) Normalized fluorescence evolution along each FZ on the corresponding topographic profile of (B) with the same color code. The thick plots correspond to FZs performed on the topographies of (A). D) Sequential topographical profiles after executing the corresponding FZs with an Si_3_N_4_ tip on an intact particle (Figure S4). The thick plots correspond to the topographies #1, #3, and #9 of Figure S4 out of the nine consecutives FZs. E) The normalized fluorescence evolution along each FZ on the corresponding topographic profile of (D) with the same color code. The thick plots correspond to the FZs performed on the topographies of Figure S4. F) Fluorescence evolution charts of five and eight VLPs during the gradual disruption induced by Au (red) and Si_3_N_4_ (blue) tips, respectively. Quenching *q* is defined as *q* = 1 − *f*, where *f* is the normalized fluorescence. The dotted lines correspond to the panels (B,C) and (D,E). The thick lines depict the average for Au (red) and Si_3_N_4_ tips (blue). Only the Au tip was able to quench the fluorescence of collapsed particles (10 nm in height). G) Normalized fluorescence evolution during an FZ (black chart, left axis) performed on unpacked GFP debris (Figure S6) with Si_3_N_4_ (blue) and Au (red) tips. Again, only the Au tips quenched the fluorescence of the GFP stains, analogous to the collapsed particles.

The intact VLP of Figure 4A (#1) was consecutively probed ten times with an Au tip and subsequently imaged following each probe. The topographical profiles obtained at the center of the particle demonstrate that its structure was gradually demolished during the process (Figure 4B). The VLP was initially intact (≈60 nm in height) and kept its spherical shape until frame #2, although it did lose some height after the first FZ (Figure 4B, #2). Thereafter the structural changes induced were more profound, and material was expelled until the final image, which shows a collapsed structure that hardly reached 15 nm in height (Figure 4A, right, and Figure 4B #11). The simultaneously recorded fluorescence signal during each FZ has been color coded (Figure 4C) as the topographical profile on which the FZ was executed (Figure 4B). These data show quenching effects that decrease the fluorescence signal to ≈0.7 (Figure 4C, FZ #1 and #2) while the VLP topography showed a recognizable spherical shape (Figure 4B, profiles #1 and #2) although the height decrease is evidence of the damage incurred. It might have been assumed that when the VLP was compressed within the evanescent field, the fluorescence signal would have been greater, but the opposite was observed. When the spherical shape was lost the fluorescence did not fall below ≈0.95 (Figure 4C, #4).

#### 2.4.2. Insulator Si_3_N_4_ Tip

For the sake of completeness, we performed similar experiments with the Si_3_N_4_ tips on a different particle. The intact VLP (≈60 nm height) of Figure S4, (#1) was consecutively probed nine times with an Si_3_N_4_ tip to induce a stepwise dismantling that finished with the complete destruction of the VLP with debris all around it (Figure S4, #9). As in the case of the Au tip, the topographical profiles (Figure 4D) showed that the VLP structure was gradually disrupted after each FZ. Their corresponding fluorescence signals, likewise color-coded, (Figure 4E) also present a quenching evolution with normalized fluorescence values ranging from ≈0.9 for FZs #1 and #2 to ≈1 for FZs beyond #3. Once more, maximum quenching occurred when the VLP structure kept its spherical shape (Figure 4D, profiles #1–3 and Figure 4E, FZ #1–3). Fluorescence recovery took place after the tip was released from the VLP when using both the Si_3_N_4_ and the Au tips at *t* ≈5 s (Figure 4C,E). We can define the normalized maximum quenching *q*_max_ by subtracting the minimum normalized fluorescence from 1 (see details in Figure S5). For example, *q*_max_ (*t* = 3 s) = 1 − 0.7 = 0.3 in Figure 4C.

#### 2.4.3. Comparison of Results

The results of these experiments have been summarized in Figure 4F, by plotting *q*_max_ (ascribed to minimum fluorescence *I*_min_, Figure S5) as a function of the virus height for five and eight VLPs with Si_3_N_4_ tips (blue) and Au tips (red), respectively. The dotted graphs correspond to the cases of Figure 4A (red, Au) and Figure S4, (blue, Si_3_N_4_); the solid thick charts depict the averaged evolution for particles indented with Si_3_N_4_ tips (blue) and Au tips (red) of single particle experiments (thin lines). We also explored the fluorescence of the unpacked GFP stains (Figure S6) outside the VLP that had not been subjected to photobleaching (Figure 4G). These data show that the Au tips (red) quench the fluorescence before establishing contact with the debris structure (0 s), and recover after release (10 s). However, the Si_3_N_4_ signal (blue) remained flat, even when the tip touched the GFP debris, indicating the absence of quenching.

### 2.5. Probing P22 VLPs Packing Less GFPs

**Figure 5.**
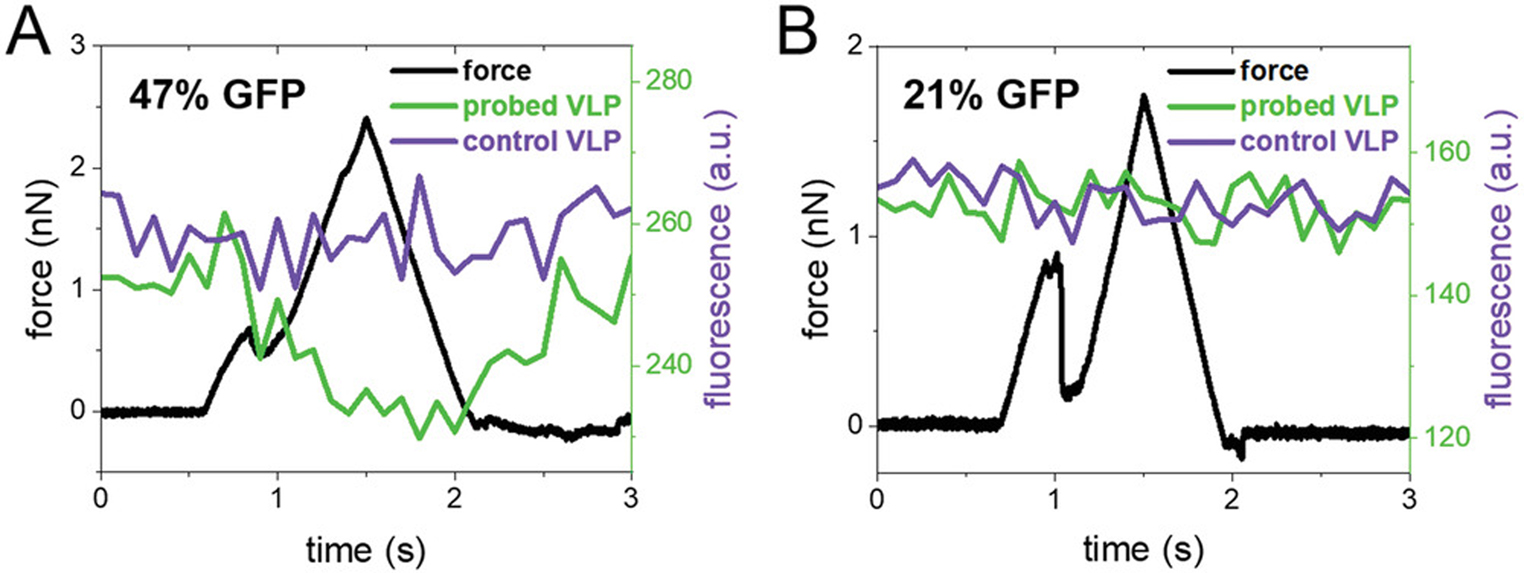
Probing mechanical quenching of VLPs with low GFP packing fraction. Simultaneous indentation and fluorescence data of A) 47% and B) 21% GFP-loaded VLPs, including the light of the control particles (Figure S7). While quenching still occurred with ≈100 GFPs inside the cage, it was suppressed when the cargo was reduced to ≈40 GFPs. The AFM images of these particles before and after indentation are shown in Figure S7.

In order to explore the influence of the number of GFPs we also performed experiments on in vitro produced VLPs^[29]^ with less fluorescent proteins than used in vivo. However, in vitro VLPs have little mechanical stability and were routinely destroyed under AFM scanning, and this hampered the experimental approach. This mechanical weakness could be due to an incomplete assembly of the VLPs which showed a few missing CPs.^[56,57]^ Nevertheless, we achieved an in vitro study using VLPs packed with 47% (*N* = 7, ≈100 GFPs) and 21% (*N* = 7, ≈40 GFPs) of the cargo loaded in the in vivo VLPs (Figure 5 and Figure S7. Both indentations showed the typical steps corresponding to capsid breakage. However, while the particles packing 47% of the GFPs loaded in vivo showed quenching (Figure 5A, green), the indentation of VLPs with a lower packing fraction was not accompanied by fluorescence quenching (Figure 5B, green). We also plotted the fluorescence data (Figure 5, purple) of the nearby control particles (Figure S7) that also produced a flattened linear behavior. These data have revealed that mechanical quenching depends on the crowding conditions of GFPs inside the P22 VLP.

## 3. Discussion

Since GFP works similarly either when packed inside VLPs or when free in solution (Figure S1), crowding is not an important factor for GFP emission. However, our data indicate that the activity of packed GFPs is sensitive to changes in their microenvironment depending on the crowding conditions.

### 3.1. Resolving the Difference between Electronic and Mechanical Quenching

**Figure 6.**
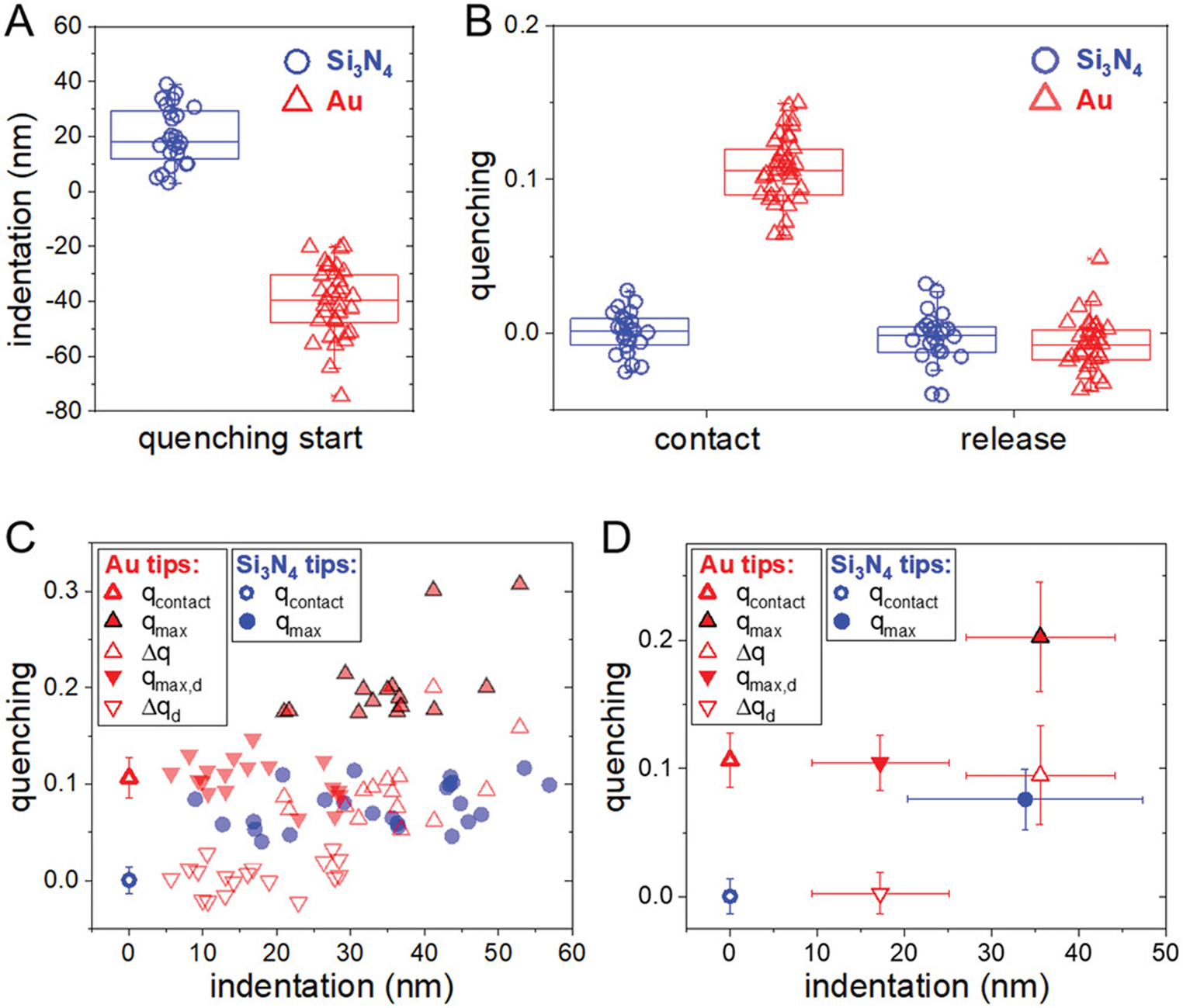
Isolating electronic and mechanical quenching. A) Indentation at which fluorescence quenching starts for Si_3_N_4_ (blue) and Au (red) tips during indentation. The positive values illustrate that the tip deforms the virus, and the negative data with an absolute value indicates the distance of the tip from the virus. Boxplots display the 25th percentile (bottom line), the median or 50th percentile (middle line), and the 75th percentile (upper line). The whiskers extend to the lowest and highest values, excluding the outliers, which are marked with a cross. B) Charts of quenching values for Si_3_N_4_ (blue) and Au (red) when the tip established contact with the particle and when it released the virus. Quenching at contact can be taken as the contribution of only electronic quenching and is evaluated as *q*_contact_ = 1 − *I*_contact_/*I*_0_, where *I*_contact_ and *I*_0_ are the fluorescence signals when the tip established contact with the particle and the value just before it started to decrease, respectively (Figure S5 ). Quenching at release is calculated similarly, but using the light signal at release instead of *I*_contact_. As described in (A), boxplots indicate the 25th, 50th, and 75th percentiles, the whiskers extend to the minimal and maximal values, and outliers are marked with a cross. C) Quenching parameters for every indentation experiment. The maximum quenching (*q*_max_) is calculated by using the minimum light signal *I*_min_ (Figure S5). For the Au tips the Δ*q* = *q*_max_ − *q*_contact_ (empty red thin triangles pointing upwards) indicate both the mechanical quenching and *q*_max,d_ the maximum quenching obtained on the damaged particles, which is similar to *q*_contact_ (empty red thick triangle pointing upwards, which is electronic quenching only). When isolating mechanical quenching on the damaged particles by Δ*q*_d_ = *q*_max,d_ − *q*_contact_, the quenching is reduced to 0 (empty triangles pointing downwards). The values of *q*_contact_ plotted at a 0 nm indentation corresponds to the median of both left boxplots in (B) and the error bars are the standard deviation. D) Plot of the median and standard deviation for each quenching parameter of (C) with the same symbols and color code. The number of particles analyzed was 24 for the Si_3_N_4_ tips and 32 for the Au-covered tips.

Our experiments have revealed that both Au-covered and Si_3_N_4_ tips alter the fluorescence signal by inducing reversible quenching when probing individual VLPs. We discuss here the differences and similarities by defining a few critical parameters. We designated the initial fluorescence *I*_0_ as the VLP intensity just before it starts decreasing. In Figure 3C for an Au-covered tip this occurs at *t* ≈1 s with an indentation of −49 nm (Figure S5). The minus sign implies that the tip is 49 nm away from the virus particle. The chart that shows the data for the insulator and metallic tips (Figure 6A) revealed that while the Si_3_N_4_ tips start quenching when the tip indents the VLP structure ≈20 nm (blue), the Au tips decrease the fluorescence well before establishing contact with the VLP surface (≈40 nm away), which suggests an electronic quenching mechanism.

If we define *I*_contact_ as the fluorescence intensity when the tip touches the VLP structure (indentation = 0 nm at *t* = 1.6 s, Figure 3C and Figure S5) our experiments did not reveal any quenching (*q*_contact_ ≈ 0) for the Si_3_N_4_ tips but the Au tips resulted in a fluorescence reduction of 12% (*q*_contact_ ≈ 0.12) (Figure 6B). These data indicate that the Au-covered tips can induce quenching at a distance, but the silicon nitride tips have to mechanically disrupt the VLPs in order to quench the fluorescence. Likewise, our data suggests that the Au-covered tips are also able to induce mechanical quenching. Figure 6C plots the quenching parameters against tip indentation for all the experiments, where *q*_max_ indicates the maximum quenching (minimum fluorescence signal *I*_min_, Figure S5) for the Au tips (solid red triangles pointing upwards) and the Si_3_N_4_ tips (solid blue circles). This figure also plots Δ*q* = *q*_max_ − *q*_contact_ (empty red triangle pointing upwards, Figure 6C,D) that can be interpreted as the residual mechanical quenching for the Au-covered tips. Figure 6D presents the processed data of Figure 6C, which shows that *q*_max_ occurs at the same indentation values for both the Au and Si_3_N_4_ tips (≈35 nm). Interestingly, in the case of the Au-covered tips, Δ*q* (empty red thin triangle pointing upwards) reaches a similar value to the Si_3_N_4_ tips, *q*_max_ (solid blue circle), thus indicating that the mechanical quenching induced by the Au and the Si_3_N_4_ tips is similar. The structural state of the VLPs also impacts the photophysical influence of the AFM tip on the VLPs. In this regard, the quenching obtained in the experiments performed with the Au-covered tips on completely damaged VLPs *q*_max,d_ (Figure 6D, solid red triangle pointing downwards) is suppressed after subtracting the electronic contribution (*q*_contact_); Δ*q*_d_ = *q*_max,d_ − *q*_contact_ ≈ 0 (empty red empty triangle pointing downwards). Therefore, as it occurs with Si_3_N_4_ tips, Au-covered tips do not induce mechanical quenching on damaged VLPs.

It is also informative to pay attention to the quenching evolution of the individual VLPs while they are being progressively damaged (Figure 4F). The averaged quenching data plotted against the VLP’s height for the Au-covered tips (solid red) rise above the value of the Si_3_N_4_ tips (solid blue) with a gap of ≈0.11. This difference is retained even when the VLP structure has been thoroughly demolished to a height of only 10 nm (see also Figure 4G and Figure S6). While the mechanical quenching induced by the Si_3_N_4_ tips only happened on intact VLPs or damaged particles that had maintained their spherical shape (Figure 4D,E), the remote quenching induced by the Au-covered tips occurs under all conditions, irrespective of the structural state of the VLP (Figure 4B,C,G and Figure S6). Therefore, the phenomenology of the Au tips helps to isolate the mechanical quenching by dispelling any other cause of quenching when using Si_3_N_4_ tips, such as electron transfer.^[58]^

### 3.2. Electronic Quenching (Au-Covered Tips)

**Figure 7.**
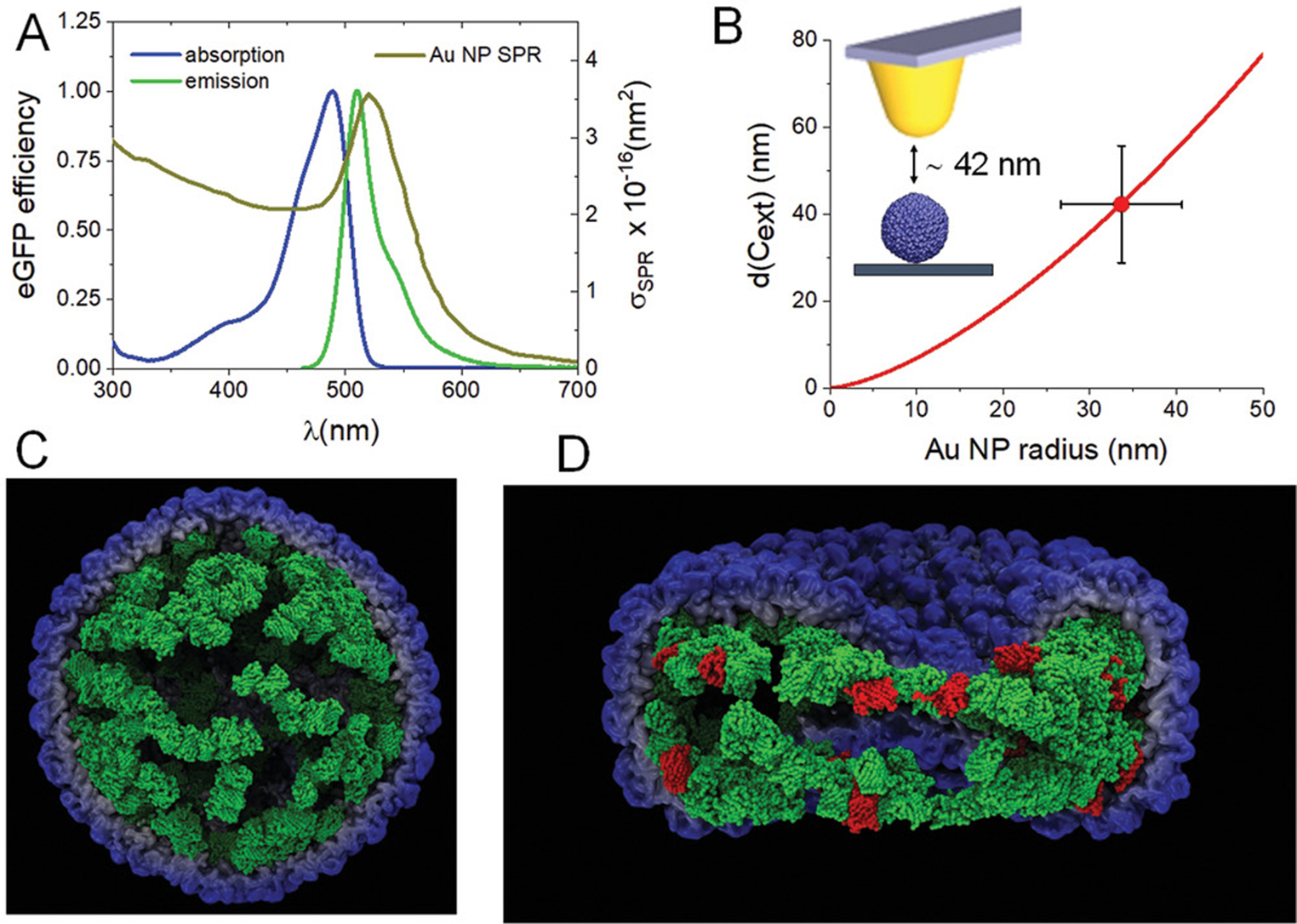
Origins of the electronic and mechanical quenching. A) The GFP efficiency and SPR cross section coefficient for an Au NP of 20 nm diameter.^[62]^ B) A plot of the cross section coefficient distance as a function of an Au NP radius. The value marked at a 42 nm distance corresponds to the median of the red boxplot in Figure 5A (start of quenching). The error bars are the standard deviation. C) A simulation (Experimental Section) of the GFP aggregates (green) inside a P22 procapsid (blue) before deformation. In this 3D image, one half of the capsid has been removed to show the internal configuration of the GFPs located below the virus lumen. D) A deformed virus where the partially unfolded GFPs are highlighted in red.

We discuss here the origin of the quenching induced from a distance by the Au-covered tips. The oscillating electromagnetic field of light induces a collective coherent oscillation of the free electrons present in an Au nanoparticle (Au NP).^[54]^ The amplitude of the oscillation reaches a maximum at a certain frequency, and this is called the surface plasmon resonance (SPR). The coupling of the Au NP’s SPR with the emissive states of fluorophores causes their quenching. This effect has been used as a ruler for building nano-optical structures,^[59,60]^ in nanomedicine^[61,62]^ as well as an aid to understanding the photophysics of the single dyes-metallic nanostructures,^[63]^ amongst other applications. It is interesting to note that the SPR wavelength depends on the size of the Au NPs.^[54]^ In particular, the absorbance peak of an Au NP of 20 nm in diameter^[61]^ is located at 520 nm (10 nm above the GFP emission peak), whereas the GFP emission efficiency is ≈0.8 (Figure 7A).

According to the manufacturer (Experimental Section and https://www.spmtips.com/afm-tip-hq-csc38-cr-au), the size of the Au covered tips is less than 30 nm, and therefore it could mimic an Au NP of 30 nm with a similar spectrum to the 20 nm diameter Au NP.^[54]^ Therefore, we propose that the Au tip SPR captures the emission of the VLP-packed GFP when it approaches the protein cage, quenching the fluorescence from a distance of 40 ± 20 nm (Figure 6A). This range of influence is not only comparable with experiments performed with real Au NPs,^[59,64,65]^ but also with previous work where the quenching of nanodiamonds attached to the AFM tip occurs at a distance of 60 nm away from an Au surface.^[66]^

The extinction cross section coefficient (*σ*_EXT_) can be roughly assumed to give a reasonable estimation of the scale of the distance of SPR influence. This coefficient has an analytical expression in the quasi-static approximation,^[54]^ when the Au NP size is much smaller than the wavelength of light:

$$\sigma_{\text{ext }}=\frac{24\pi^{2}{\text{r}}^{3}\varepsilon_{\text{m}}^{3/2}}{\lambda }\frac{\varepsilon_{\text{i}}}{{\left(\varepsilon_{\text{r}}+2\varepsilon_{\text{m}}\right)}^{2}+\varepsilon_{\text{i}}^{2}}$$

In this expression, *λ* is the wavelength of the incident light (488 nm), *ε* is the complex dielectric constant of the metal given by *ε* = *ε*_r_ + *iε*_i_, *ε*_r_ and *ε*_i_ are the real and imaginary parts of the dielectric function of Au; *ε*_m_ is the dielectric constant of the surrounding medium (H_2_O) and *r* accounts for the radius of the Au NP. Since we know that quenching starts when the Au-covered tip is ≈42 nm away from the VLP (Figure 6A), we can assume that this distance corresponds to a certain *σ*_EXT_. By plotting the size d(*σ*_EXT_)^[61]^ as a function of the Au NP radius *r* (Figure 7B) we find that for d(*σ*_EXT_) = 40 ± 20 nm the Au NP size should be 33 ± 7 nm, which compares favorably with the radius of a tip that has been worn out during the experiment.^[67]^

The electronic quenching only reaches ≈10% of the total fluorescence signal (Figure 6D, Δ*q* and *q*_contact_). This partial quenching might indicate that only a few of the ≈209 packed GFPs are affected. This effect could be related to the fact that the geometry of the tip controls the scope of SPR because the spherical-like tip apex enhances the SPR effect^[68]^ but we had been using a parabolic tip apex in this experiment. Also, because the Au-covered AFM tips are not solid metal, unlike the Au NPs^[54]^ this might have reduced the affectivity of the SPR coupling.

### 3.3. Mechanical Quenching (Si_3_N_4_ Tips)

Our data indicate that mechanical quenching is necessarily induced by the geometrical alterations of GFP molecules that occur during the breakage of the VLPs. When the VLPs yield under the mechanical force of the AFM tip (Figure 3A) the GFPs, which are bound to the VLP lumen through the SP (Figure 1), are subjected to alterations in their relative position. In particular, in the wall-to-wall collapsed virus there is a sudden approach between GFPs (Figure 7C) at subnanometer distances. The close proximity of fluorescence molecules might induce homo-Förster Resonance Energy Transfer (FRET) between the GFPs.^[69]^ In the case of GFP, the adsorption and emission peaks (Figure 7A) are close enough to enable this effect.^[70]^ However, our experiments have monitored the total fluorescence signal in all the polarization planes, and this was not affected by homo-FRET.^[71]^ In addition, another method of assessing whether the decrease in fluorescence occurred as a result of FRET is to reduce the density of active GFPs in situ.^[72]^ This should increase the distances between active fluorophores in the collapsed capsid thereby reducing FRET efficiencies. In this study, we bleached the VLPs until they reduced their emission by 50% and found that the mechanical quenching was similar to the unbleached particles (Figure S8). Therefore, homo-FRET can be rejected as the main factor responsible for mechanical quenching.

A second scenario to be considered is the structural integrity of the GFPs during VLP collapse. Optical tweezers experiments have reported that GFP switches off a few milliseconds before the first unfolding event.^[40]^ During this stage, which is induced by a force of 35 pN, a portion of the short N-terminal α-helix consisting of residues 1 to 6 had been pulled away from the barrel^[73]^ with an energy cost of 22 *k*_B_*T*.^[74]^ As a consequence, there was no intermediate fluorescence emission from the dark to the emissive estates during the unfolding process of GFP revealing that protein integrity is crucial for fluorescence emission.^[40]^ To investigate this further we revised the microenvironment of the packed GFP inside the P22 procapsid. Fluorescent proteins are prone to form oligomers under high concentrations^[75]^ and GFP in particular exhibits several oligomerization states, including dimers, trimers, and tetramers.^[76]^ In the P22 prohead system there are ≈209 GFPs^[41]^ attached to the capsid lumen with an internal radius of 22.2 nm, resulting in a nearest neighbor distance of ≈3 nm between the protein centers. Since the GFP Stokes radius is 2.8 nm, we derived an average distance of 2 Å between fluorophores (Figure 1A). It is likely the high packing condition of GFP on the lumen of the capsid favors the aggregation of proteins (Figure 7C).^[76]^ GFP aggregation has been previously described at high concentrations, when it was used to label the plasma membrane domain^[77]^ and impedes their fusion with proteins of interest.^[75]^ We have found indications of GFP aggregation in previous experiments by analyzing the cargo retention of P22 VLPs.^[78]^ In the structure of P22 “wiffle ball”, GFP is unbound from the virus lumen and the icosahedral capsid lacks pentamers, resulting in 12 pores each with a diameter of 10 nm. As a consequence, while GFP oligomers smaller than this can escape from the VLP, the larger aggregates remain trapped inside. Cargo retention experiments in the P22 “wiffle ball” revealed that 1/3 of GFPs were trapped inside the VLP cavity, suggesting that this cargo was aggregated into oligomers with a minimum size of 10 nm.

Another indication of GFP aggregation is provided by an energetic analysis of the system structure. The binding energy of GFP dimerization (45 ± 16 *k*_B_*T*)^[79]^ is approximately twice larger than the energy of the first GFP unfolding event (22 *k*_B_*T*) and almost ten times the energy of CP-CP binding (≈10 *k*_B_*T)*.^[80]^ The energy applied by the tip in a typical indentation experiment that breaks a protein shell (Figure 3B) is ≈200 *k*_B_*T*.^[81]^ In addition the cryo-EM reconstructions of P22 procapsids loaded with GFP unveiled the organization of packed cargo molecules as a subshell beneath the P22 CP capsid.^[24]^ We have also reported the mechanical experiments performed on the P22 procapsids which indicated that the GFP located at the lumen increases the virus stiffness by ≈78% compared with the empty capsid. Finite element modeling suggests that this increment accounts for the GFPs forming a homogeneous shell below the virus lumen that is compatible with the local aggregation of GFPs. In fact, the increase in mechanical stability, induced by the same amount of GFP in expanded P22 particles,^[24]^ where the proteins detach from the virus lumen, is only ≈30%.^[24]^ These energetic and mechano-structural considerations indicate that during the indentation of the P22 nanocontainer the GFP subshell will yield at the weakest interactions. Theoretical simulations of this deformation (Experimental Section) show that some GFPs undergo unfolding (Figure 7D, red), evolving from the emissive to the dark state and resulting in the partial quenching of the VLP particle.

Interestingly, mechanical quenching is precluded when the indented GFP proteins are released from the VLPs and/ or collapsed particles (Figure 4G). The structural situation of unpacked GFP in collapsed particles is different: GFPs bound to small pieces of the virus protein shell rest on the surface in the absence of any geometrical constraint or structure. In these circumstances, the mechanical contact with the AFM tip cannot induce relevant deformations to the GFPs. This idea can be imagined by thinking of the influence of geometry on the mechanical resistance of a macroscopic material. Cardboard, for example, can be easily punctured and collapsed when it is folded forming a box, which is what happens with an intact P22 particle. However, the same cardboard unfolded and resting on a hard surface is much more resistant to mechanical stress, as seen with the hardly damaged particles (Figure 4). In our system, this effect is further illustrated by the comparison in Figure S9 of the force-indentation data of intact particles (Figure 3C, black) and collapsed particles/GFPs (Figure 4G, black). After establishing contact with the virus, the indentation curve performed on the intact particles (Figure S9, gray) reflects a linear increase in the force until initiating the breaking of the particle at 6 nm of indentation. At this point, the fluorescence (light green) starts to decrease. Following this, there are a succession of steps that indicate the breakage and collapse of the virus until almost 55 nm, where the tip reaches the stiff surface. The fluorescence signal (light green) continues to reduce during this process. In the case of the indentation on the unpacked GFP debris (Figure S9, black), data hardly show a Hertzian deformation of less than 5 nm before the infinity slope of the force indicates the hard surface of the substrate. In this case, the fluorescence (dark green) remains unaltered during the experiment. From a comparison of both curves, it is evident that GFP unfolding is difficult when it is not framed by the virus lumen. The cardboard analogy also helps to understand why the quenching does not increase in general with indentation (Figure 6C,D). Once the VLP yields through certain parts inducing partial unfolding of some GFPs and particle collapse, a larger indentation does not make the particle break in more places. If the cardboard box (virus cage) yields through the edges, it will just collapse on itself without deforming any other sites, no matter how much it is compressed.

We suggest that the geometrical constraints imposed by the VLP cavity on the highly packaged GFP induce aggregation and concentrate mechanical stress on transiently unfolding some GFPs. Since mechanical quenching reduced the total fluorescence signal by ≈10% (Figure 6D), it can be assumed that only ≈20 GFPs were affected during capsid breakage (Figure 7D). Our hypothesis also endorses the fluorescence recovery observed once the tip released the protein cage, since the partially unfolded GFPs refold if the VLP structure recovers its spherical shape^[52]^ (Figure 4).

What is the influence of crowding in the GFPs emission? Molecular crowding has two major effects: 1) to modify the dynamics of protein conformational changes due to excluded volume effects; and 2) to favor protein aggregation that can affect their function. In vivo encapsulation of GFP in P22 VLPs already induced molecular crowding of these fluorescent proteins. However, their light emission was minimally affected (Figure S1). While crowding is irrelevant for electronic quenching, the aggregation of crowded GFPs inside the VLP cavity resulted in a decrease in fluorescence when the particle was broken under mechanical stimuli. In fact, when the quantity of GFPs inside the VLP cavity is reduced to 21% of the in vivo value (Figure 5B) mechanical quenching does not occur, indicating that aggregation did not take place. Therefore, our experiments mainly probe the effect of crowding on the GFPs sensitivity to changes in the mechanical microenvironment due to their aggregation.

## 4. Experimental Section

### P22 Production Methodology:

A pRSF-Duet vector containing the CP and GFP-SP genes in MCS (multiple cloning site) 1 and 2, respectively, was transformed into BL21 Electrocompetent Cells. Both genes were expressed on LB medium at 37 °C with kanamycin to maintain selection for pRSF-Duet. After the cells reached OD_600_ 0.5–0.7, the growth was induced with 13.3 mm arabinose and incubated for an additional 4 h at the same temperature. The culture was then centrifuged to harvest the cells and resuspended in a lysis buffer (pH 7.0, 50 mm sodium phosphate buffer, 100 mm sodium chloride). After one freeze-thaw cycle, the cells were lysed by incubating with lysozyme, DNase, and RNase for 30 min at room temperature, followed by two rounds of sonication. The cell debris was then removed using centrifugation at 12000 × *g* for 45 min, and the resulting supernatant was ultra-centrifuged through a 30% w/v sucrose cushion at 215619 × *g* for 50 min. The resulting pellet was resuspended in 50 mm sodium phosphate buffer and 100 mm sodium chloride (pH 7.0) and purified over S-500 Sephadex column using BioRad Biologic Duoflow FPLC. The fractions containing the GFP encapsulated P22 VLPs were pooled and further purified using a 12.5–52% w/v sucrose gradient.

### Sample Preparation for AFM-TIRFM:

Glass coverslips were cleaned by sonication in a 9 m solution of KOH with 30 mL ethanol for 20 min, then sonicated three times in fresh milli-Q water for 20 min and dried in an oven at 120 °C for 10 min after clearing the excess water with gaseous nitrogen. The clean glasses were left overnight in a sealed bucket containing 2 mL hexamethyldisilazane for silanization by vapor deposition. 40 μL of a 1:400 dilution of the purified P22 VLPs in pH 7.0, and 50 mm sodium phosphate buffer with 100 mm sodium chloride were incubated for 10 min on a silanized glass coverslip and rinsed three times with 100 μL of the same buffer. Additional buffer was pipetted to reach a final volume of 100 μL.

### AFM Imaging and VLPs Manipulation:

This combined home-made AFM-TIRFM setup consisted of an AFM head placed on top of the PI-P-733.3DD piezo stage, which surrounded the TIRF objective. The coupled microscope was placed in a custom-made isolation box on top of a spring-loaded vibration cancelling system. All the heat and noise-generating parts, that is, the power supplies, camera, and laser, were mounted outside the isolation box and were mechanically decoupled from the AFM. Rectangular cantilevers RC800PSA (Olympus, Japan) and gold-coated tips HQ:CSC38/CR-AU (MikroMasch, Bulgaria) with a nominal spring constant of 0.05 N m^−1^ were used in jumping mode.^[82]^ In this mode the AFM topography image was constructed by performing a low force curve (<100 pN) in each pixel, moving the piezo laterally when the tip was out of contact to avoid dragging forces. For locating particular VLPs, an AFM image of 5 μm × 5 μm was scanned and a single TIRF-image centered at the AFM tip apex was recorded by flashing a blue excitation laser for 0.1 s in order to avoid photobleaching as much as possible. Using this method it was possible to look for coincident VLPs patterns in both AFM and fluorescence images (Figure 2 and Figure S1). Once the virus had been identified within a fluorescence pattern, the magnification was increased and a force curve (FZ) performed at the very top at a velocity of 100 nm s^−1^ (Figure 3). The excitation laser was switched on during the FZ and the simultaneous fluorescence signal was recorded (Video SV1, SI). The subsequent topographical image obtained provided information about the integrity of the VLP following FZ. This experiment was performed consecutively on the same particle until the structure finally collapsed (Figures 4 and 5).

### TIRF Imaging:

The inverted optical microscope was custom-built around a TIRF objective (100x, NA 1.49, Nikon Instruments, Japan) mounted on a Piezo LEGS motor (Piezomotor, Sweden) to focus the sample plane precisely. For fluorophore excitation a laser diode (Nichia, Japan) of 488 nm wavelength was used. Dichroic and emission filters, optimized for GFP, were obtained from Semrock (USA), and all optomechanical parts and lenses were manufactured by Thorlabs (USA). Fluorescence intensity was recorded using a Neo 5.5 sCMOS camera (Andor, UK) at an exposure time of 0.1 s.

For the fluorescence data analysis, the mean intensity of a 7 × 7 pixels^2^ ROI enclosing the particle of interest was considered. As shown in the Figure S3, any photobleaching effects were corrected by exponentially fitting the average over time of 15 ROIs (corresponding to 15 intact VLPs surrounding the particle hit by the AFM tip) and setting it constant to its initial value by adding the correction function *A* × (1 − exp(−*t*/*B)*), where parameters *A* and *B* were the amplitude and the decay rate of the exponential fit, respectively.

### Simulation of Virus Deformation:

For the molecular dynamics simulations presented in Figure 7, a coarse-grained model was used. In this model, an alpha-carbon representation was implemented, where each amino acid corresponded to a bead in the simulation.^[83]^ The mass of each amino acid was the sum of the masses of its components and the charge was set to the values expected for pH 7: +e for Lys and Arg, −e for Asp and Glu, and +0.5e for His, where e was the charge of the electron.

The model was split into two parts,^[84]^ one for the internal representation and the other one for the protein–protein interactions. For the internal part, the self-organized polymer model^[85]^ was used which had been used previously to study both the GFP’s mechanical properties^[86]^ and the viral capsid’s deformations.^[87]^ Regarding the GFPs and the viral capsid, a homogeneous parameter *ε* = 1 kcal mol^−1^ was applied for the interaction energy of the native contact. In the special case of the unstructured linker zones between the GFP and the virus capsid, in addition to the bond potential between the consecutive amino acids, angular and dihedral potentials were also considered.^[83]^

For the protein–protein interactions^[84]^ the Kim–Hummer model was used.^[83]^ As initial conditions, it was considered that the GFPs were anchored through the linker to the positions inside the virus lumen.^[24]^ Langevin dynamics simulations were run with a time step of 15 fs and a friction coefficient of 0.2 ps^−1^.^[84]^ The system was allowed to reach equilibrium (Figure 7C) and then performed the compression of the VLP between two parallel plates^[88]^ until their separation was reduced to 30 nm (Figure 7D).

## Supplementary Material

Video

Supporting Info

## Data Availability

The data that support the findings of this study are available on request from the corresponding author. The data are not publicly available due to privacy or ethical restrictions.
