# Supplementary material for: Electromechanical Photophysics of GFP Packed Inside Viral Protein Cages Probed by Force-Fluorescence Hybrid Single-Molecule Microscopy
Source: Small. Author manuscript; Available in PMC 2022 Oct 3. (PMC9528512; doi:10.1002/smll.202200059)
Supplement: Supporting Info [file NIHMS1830720-supplement-Supporting_Info.pdf]

NANO small MICRO

Supporting Information

for *Small,* DOI: 10.1002/smll.202200059

Electromechanical Photophysics of GFP Packed Inside Viral Protein Cages Probed by Force-Fluorescence Hybrid Single-Molecule Microscopy

Klara Strobl, Ekaterina Selivanovitch, Pablo Ibáñez-Freire, Francisco Moreno-Madrid, Iwan A. T. Schaap, Rafael Delgado-Buscalioni, Trevor Douglas, and Pedro J. de Pablo**

Electromechanical photophysics of GFP packed inside viral protein cages probed by force-fluorescence hybrid single-molecule microscopy

K. Strobl, E. Selivanovitch, P. Ibañez-Freire, F. Moreno-Madrid, I. Schaap, T. Douglas and P. J. de Pablo

SUPPORTING FIGURES

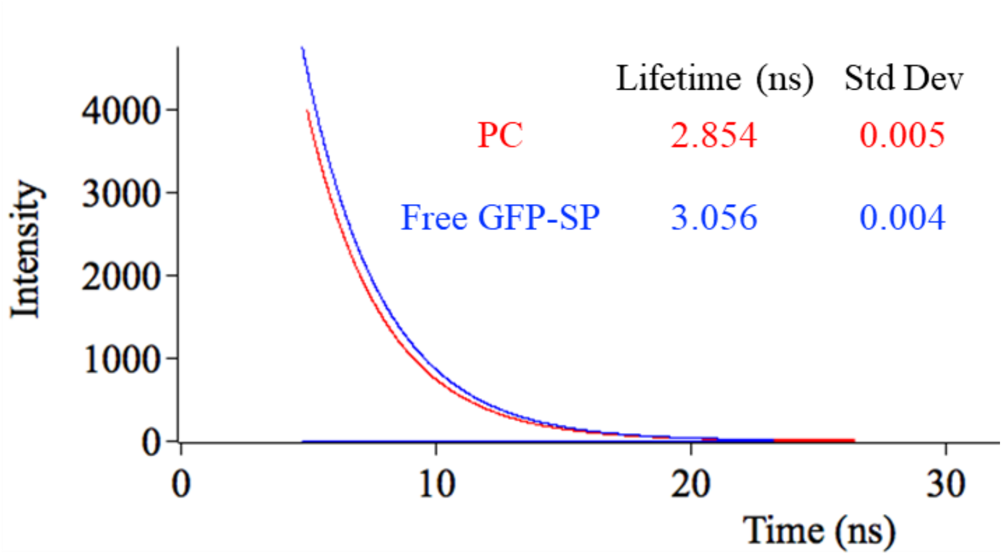

Figure S1. Measured fluorescence lifetime of P22 GFP and free GFP-SP eGFP shows that eGFP fluorescence remains unaltered after packing.

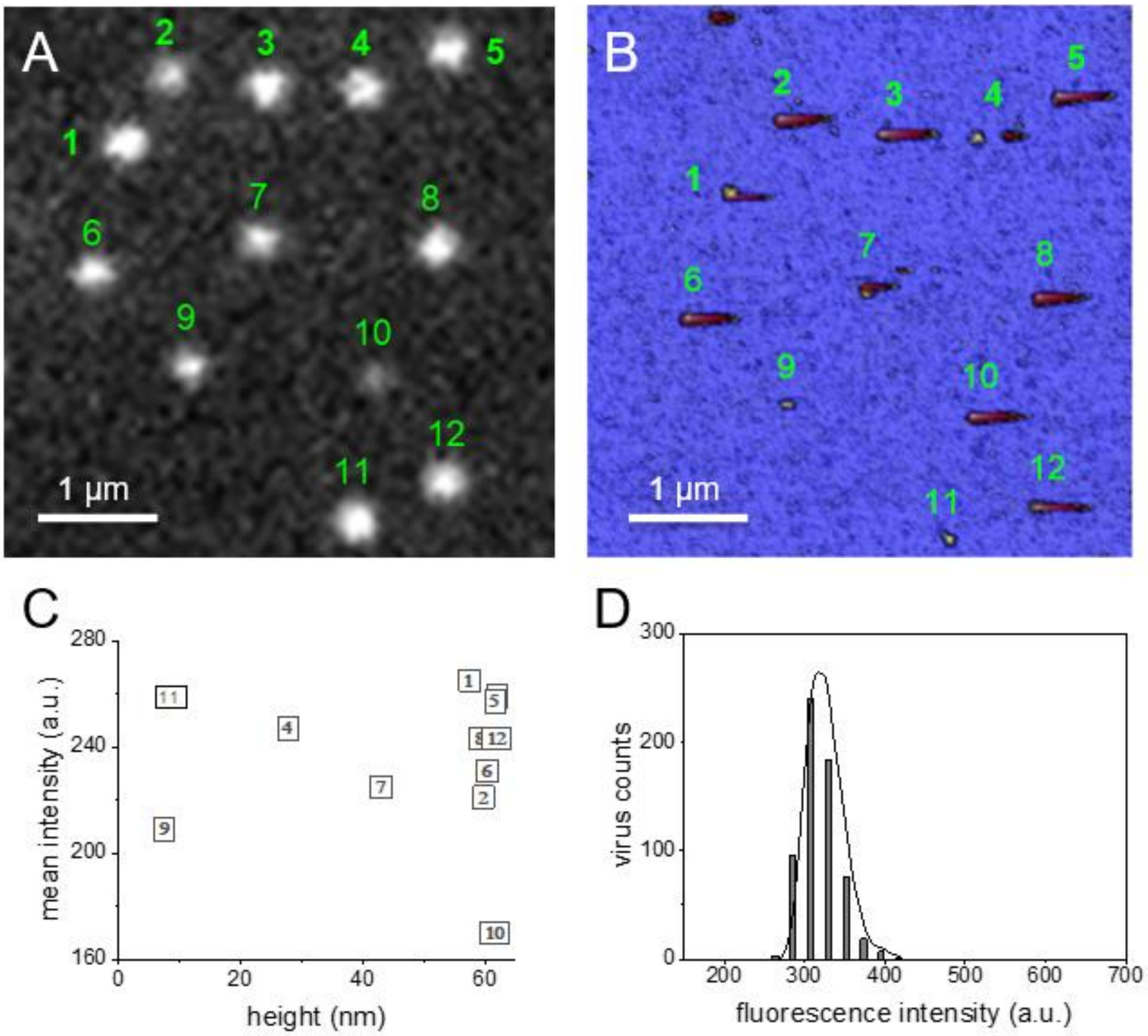

Figure S2. (A) Fluorescence image of a large area (25 µm2) with 12 labeled particles, whose correspondent AFM image (B) is equally labeled. The elongation of the particles at the right side is caused by the parachuting of the tip at the downhill part of the virus when scanning from left to right. (C) Plot of fluorescence signal vs. VLP size (height) of (A) and (B). (D) Fluorescence counts histogram of P22 VLPs.

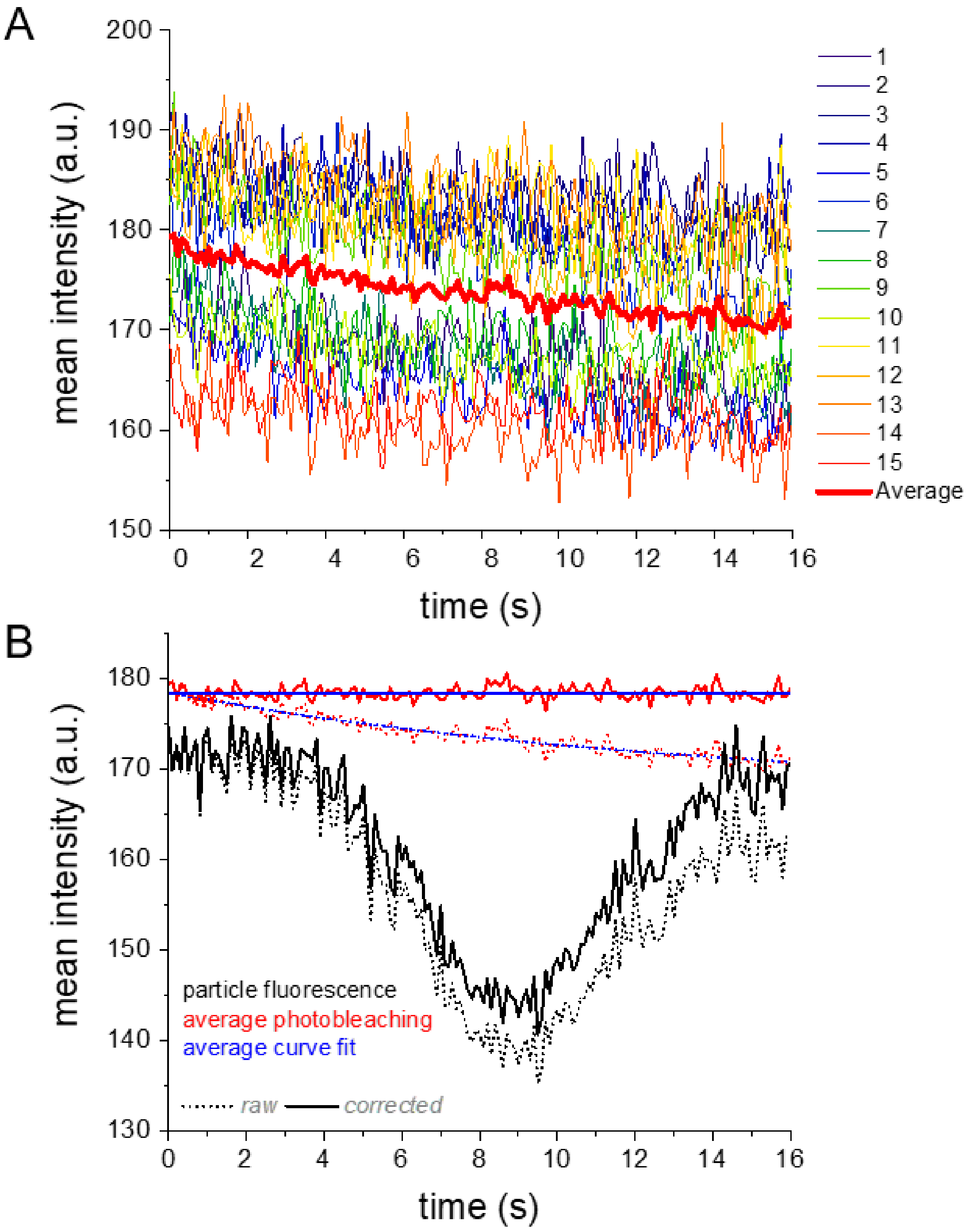

Figure S3. (A) The intensity loss due to photobleaching during indentation can be estimated from the averaged fluorescence emission of numerous particles (here 15 ROIs) surrounding the VLP hit by the AFM tip. (B) This average photobleaching can be fitted to an exponential decay (dotted blue), and can be corrected to remain constant (solid blue). The correction function that annihilates photobleaching contribution is expressed as A*(1-exp(-t/B)), where parameters A and B are the amplitude and the decay rate of the exponential fit, respectively. This contribution is added to the raw data of the hit particle (dotted lines) to result in the corrected signal (solid line).

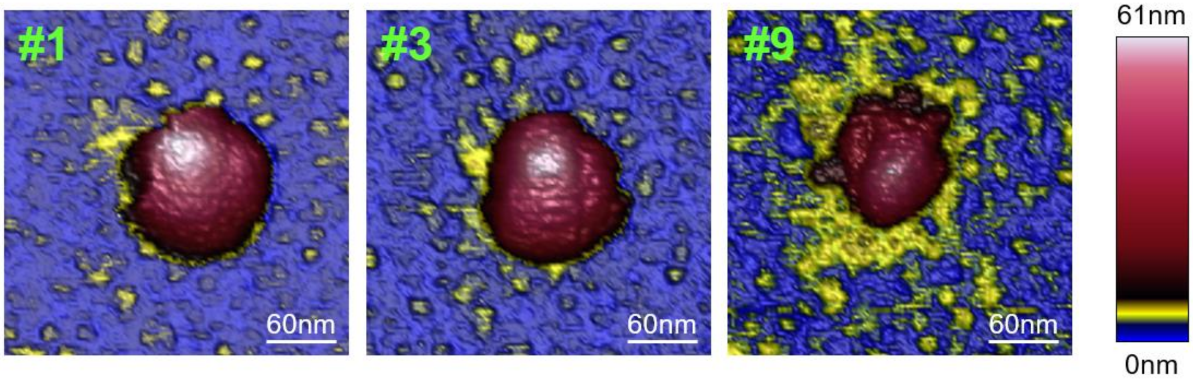

Figure S4. VLP P22 images of a particle successively indented by a Si_3N_4 tip, whose fluorescence and topographical data are shown at Figure 4D,E.

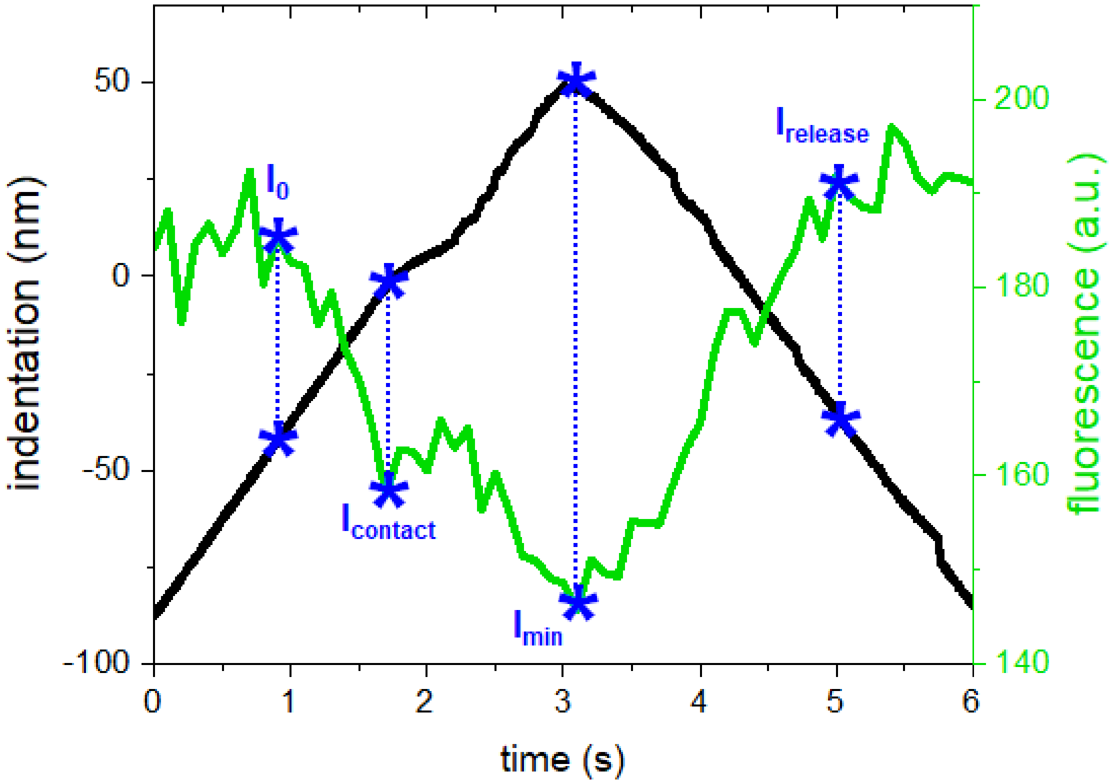

Figure S5. Plotting simultaneously the indentation and quenching of Figure 3D for extracting experimental parameters. Indentation is calculated as explained elsewhere (black)[80] and indicates the VLP deformation along the FZ experiment. The absolute value of the negative indentation indicates the distance of the tip to the VLP before contact (indentation = 0nm). In order to calculate the quenching q, we define the initial fluorescence I_0 as the intensity just before it starts decreasing (time = 0.9s, I_0 ~185 and indentation -43 nm). In this case the indention value is telling us that quenching starts when the tip is 43 nm away from the VLP, while approaching. We use I_0 as a normalization factor because this is the maximum intensity that the VLP can provide along the experiment. We calculate quenching at contact as $q_{contact} = 1 - I_{contact} / I_0$ 0.12 (t = 1.7s and indentation = 0nm). q_{max} 0.21 holds for the maximum quenching (t=3.1s) happening at minimum intensity I_{min} ~146. $q_{release}$ ~ 0 designates the final quenching value, once the AFM tip has released the virus particle (t=5 seconds, $I_{release}$ ~191).

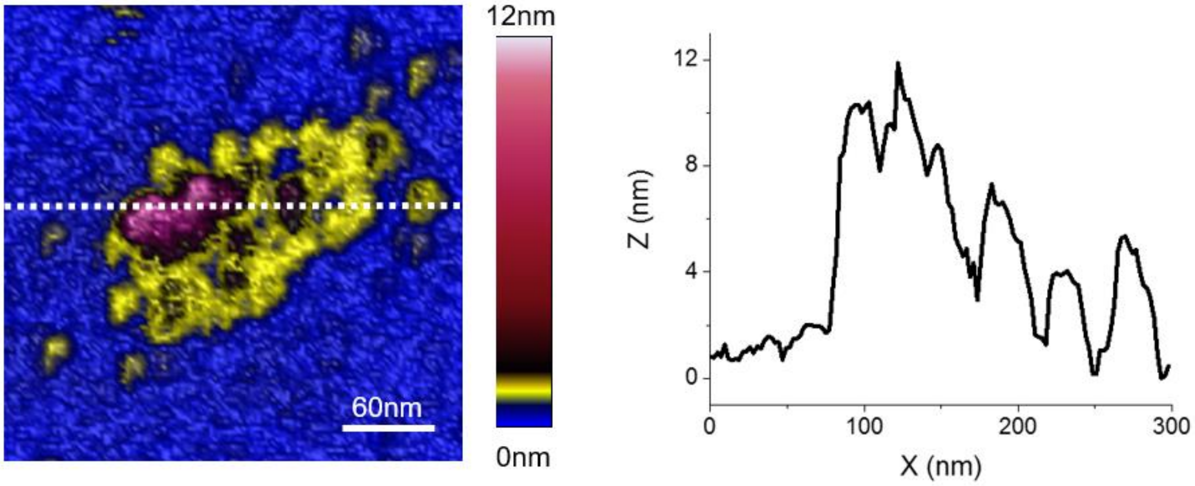

Figure S6. AFM image of eGFP stain on the surface (left). Topographical profile (right) at the white dotted line of the AFM image. Complement to Figure 4G.

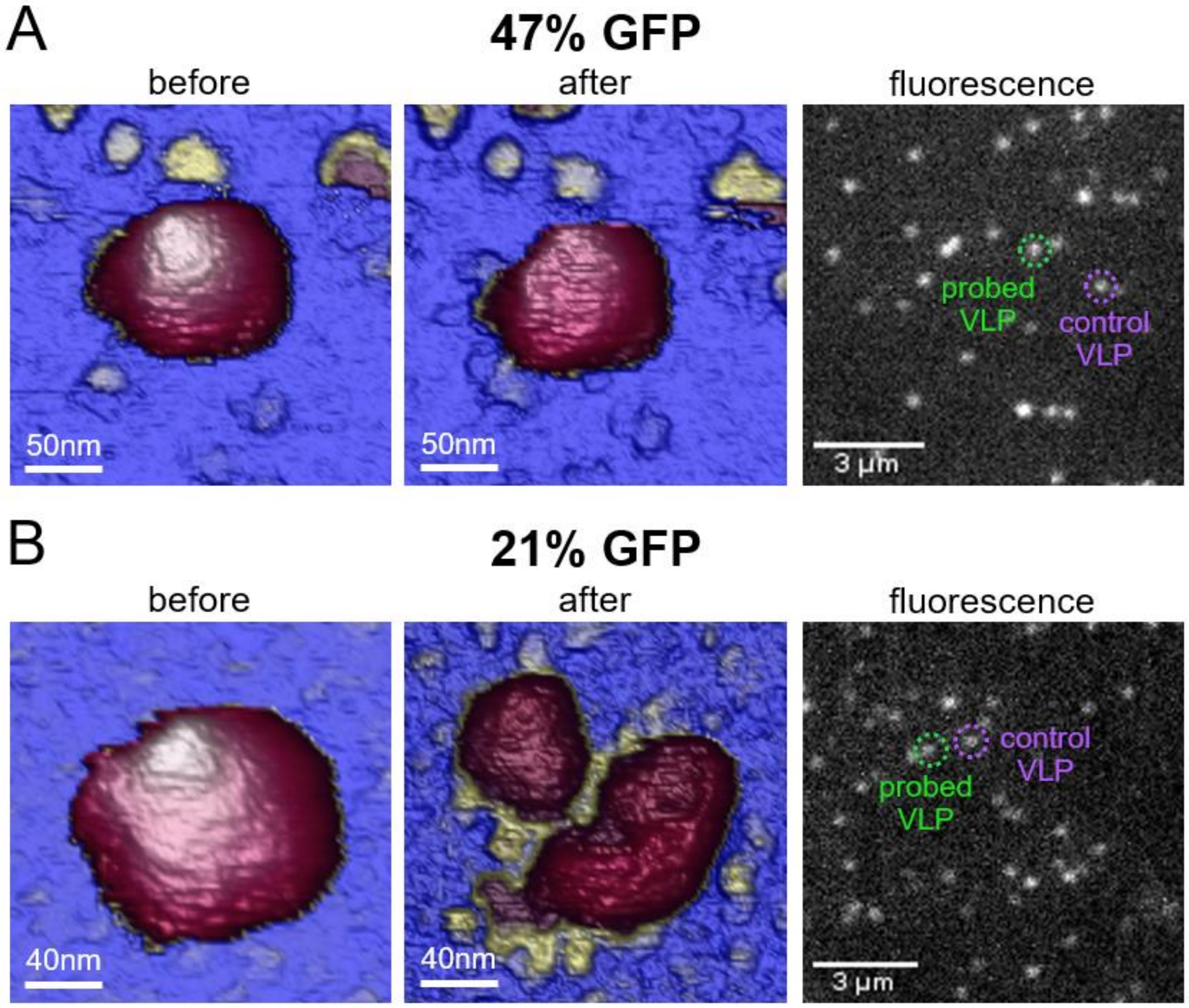

Figure S7. Complement to Figure 5. Panels A and B, left and center, show the AFM images of the VLPs before and after the indentation. The color scale used in the AFM images corresponds to the one displayed in S4. Right figures present the fluorescence images showing the probed and the control VLPs inside green and purple circles, respectively.

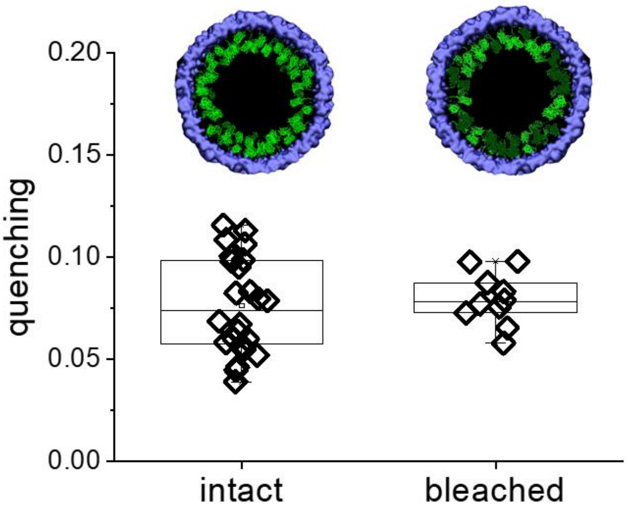

Figure S8. Quenching comparison between intact and bleached VLPs for checking possible homoFRET effects. The boxplots represent the 25th percentile (bottom line), the median (middle line) and the 75th percentile (upper line). The mean is denoted as a hollow square and the whiskers extend to the minimal and maximal values. The number of particles analyzed is 24 intact VLPs and 10 photobleached VLPs.

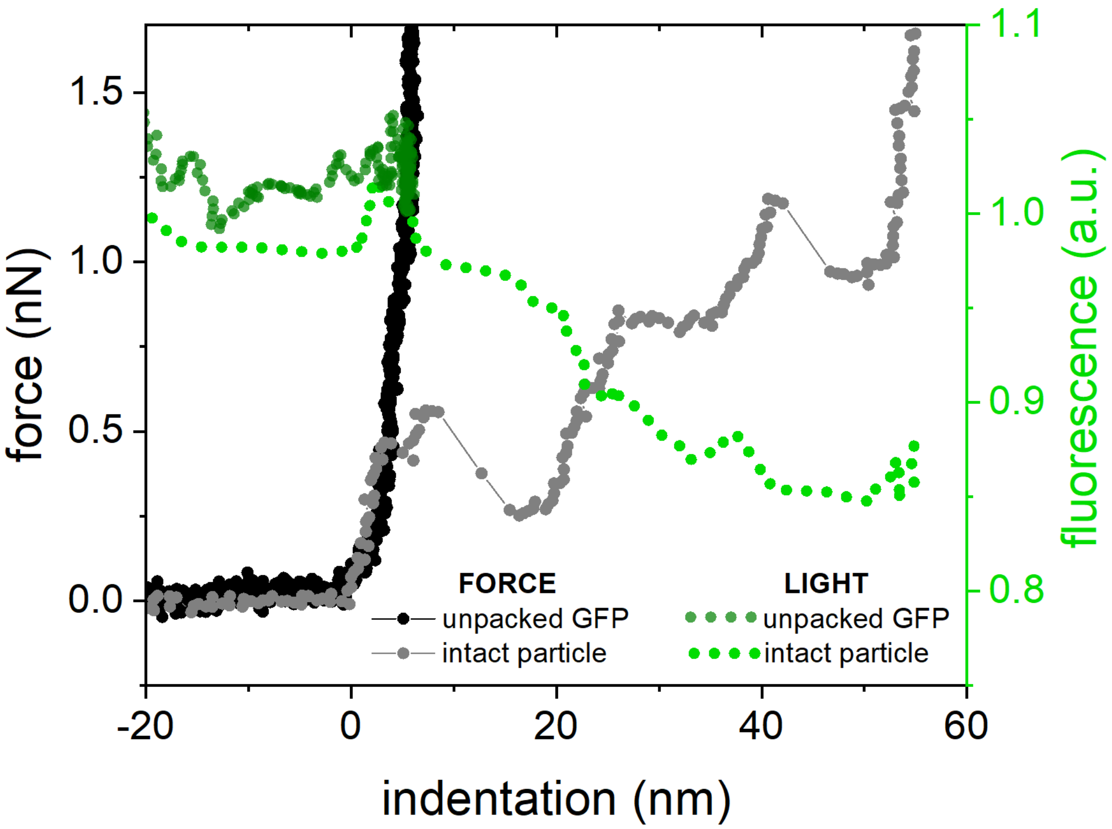

Figure S9. Force-indentation (deformation) data with their respective fluorescence signal curves for intact and collapsed viruses in dark and green colors, respectively. It is possible to observe that in the case of unpacked GFP (dark green) fluorescence remains unaltered, no matter the force. However, the emission of the intact particle (light green) decreases as the virus is indented.
